# Coagulation profile of Sudanese children with homozygous sickle cell disease and the effect of treatment with omega-3 fatty acid on the coagulation parameters

**DOI:** 10.1186/s12878-017-0089-5

**Published:** 2017-11-09

**Authors:** Shiekh Awoda, Ahmed A. Daak, Nazik Elmalaika Husain, Kebreab Ghebremeskel, Mustafa I. Elbashir

**Affiliations:** 1 Department of Medical Biochemistry, Faculty of Medicine, University of Khartoum, Alghasr Street, Khartoum, Sudan; 2 Center of Molecular Biology and Biotechnology (CMBB), Florida Atlantic University (FAU), Boca Raton, USA; 3 College of Medical Laboratory Sciences, Sudan University of Science& Technology, Khartoum, Sudan; 4 Lipidomics and Nutrition Research Centre, London Metropolitan University, London, UK

**Keywords:** Sickle cell disease, Coagulation, Omega-3 fatty acids, D-dimer, Protein C, Protein S

## Abstract

**Background:**

It has been reported that patients with SCD do have an abnormal coagulation profile. Coagulopathy is thought to be one of the key factors that contribute to the vaso-occlusive crisis that characterises sickle cell disease (SCD). In this study, we investigated whether Sudanese sickle cell patients have an abnormal coagulation profile. In addition, the effect of treatment with either omega-3 fatty acids or hydroxyurea on coagulation profile was assessed.

**Methods:**

Homozygous SCD patients untreated (*n* = 52), omega-3 treated (*n* = 44), hydroxyurea (HU) treated (*n* = 8) and healthy (HbAA) controls (*n* = 52) matched for age (4–20 years), gender and socioeconomic status were enrolled. Patients on omega-3 fatty acids, according to age, received one to four capsules containing 277.8 mg DHA and 39.0 mg eicosapentnoic. Patients on Hydroxyurea were in on dosage more than 20 mg/kg/day. The steady state levels of the coagulation parameters and the effect of the treatments with either HU or omega-3 fatty acids on markers of coagulation were investigated.

**Results:**

Compared to the healthy controls, treated and untreated HbSS patients had lower hemoglobin, plasma Protein C, proteins S and higher white blood cell count (WBC), platelets count (PLTs) and plasma D-dimer levels,(*p* < 0.05). In comparison to untreated HbSS, treatment with neither omega-3 nor HU had effect on the WBC, plasma proteins C and S, (*p* > 0.05). HU treated group had a lower PLTs count compared to HbSS untreated group (*p* < 0.5). The prothrombin and activated partial thromboplastin times and international normalized ratio (INR) of untreated patients are significantly higher than n-3 treated, HU-treated patients and health controls, (*p* < 0.05). Patients treated with omega-3 had lowered D-dimer levels in comparison to HU-treated and untreated HbSS patients, (*p* < 0.001).

**Conclusion:**

This study provides evidence that Sudanes patients have abnormal coagulation profile and treatment with either HU or omega-3 fatty acids might partially ameliorate SCD-associated chronic coagulopathic state.

## Background

Chronic hypercoagulable or prothrombotic state is generally known to be one of the factors that contribute to vaso-occlusion and progressive end-organ damage in sickle cell disease (SCD) [1, 2]. Studies on SCD patients at steady state patients from different geographic and demographic origins have shown elevated level of markers of coagulation activation [3, 4], and decreased natural anticoagulant proteins [5, 6]. Agents that physiologically activate platelets and coagulation in vivo include adenosine diphosphate (ADP), collagen, epinephrine**,** thrombin, serotonin, arachidonic acid and thromboxane A2 [7]. Interestingly, blood cell membranes of patients with SCD have an abnormal fatty acid profile which is characterised by low levels of omega-3, namely eicosapentaenoic (EPA) and docosahexaenoic acids (DHA), and a high level of omega-6, particularly arachidonic acid (AA) [8, 9]. A high intake of omega-3 fatty acids is inversely associated with thromboxane level and prothrombotic activity [10, 11]. Consequently, it has been postulated that regular intake of n-3 fatty acids in patients with SCD may modulate markers of coagulation.

On the other hand, Hydroxyurea (hydroxycarbamide) is currently the only approved drug to prevent the acute complications of the disease [12], and has several well-defined beneficial effects that might involve mitigation of the hypercoagulability state in patients with SCD [13]. In this study we investigated whether (1) Sudanese sickle cell patients have an abnormal profile of coagulation parameters; (2) the coagulation parameter are modified by omega-3 fatty acids or hydroxyurea.

## Methods

### Subjects

In this study, steady state homozygous sickle cell patients (HbSS) on high DHA omega-3 treatment (*n* = 44), hydroxyurea treatment HbSS (HU, *n* = 8), steady HbSS patients not treated either with HU or omega-3 fatty acids (*n* = 52) controls and healthy sibling controls (HbAA, *n* = 52), matched one to one by age (4–20 years), gender and socioeconomic status were included. “Steady state” is defined as being free from acute painful crisis or other medical condition for at least one month before the enrolment. The patients and healthy controls, who were mostly siblings of the patients, were enrolled from Abnaof Paediatric Hospital, Khartoum, Sudan, between February and May 2014. We selected the patients’ healthy HbAA siblings as controls in order to minimise the potential effect of dietary background and genetic factors on the variables under investigation. Haemoglobin phenotypes were confirmed using cellulose acetate electrophoresis at pH 8.5. The exclusion criteria for participation in the study were other chronic disorder and conditions (defined as the one that lasting 3 months or more), blood transfusion in the previous four-months. The patients on n-3 fatty acids received a daily dosage of one, two, three or four capsules according to the patient’s age (< 5), (5–10), (11–16) and (>17) years, respectively. The n-3 capsule contains 277.8 mg docosahexaenoic (DHA) and 39.0 mg eicosapentnoic (EPA) and (1.5 mg) of vitamin E to prevent fatty acids peroxidation. The n-3 fatty acid group was on same dosage of n-3 fatty acids for minimum of three years. The hydroxyurea group was treated daily with a dose of 20 mg per kilogram body weight orally for minimum of at least one year. The patients participated in this study were on regular folate supplement. The healthy control subjects did not receive folate or any other nutritional supplement.

The equation (n = (Z_α/2_ + Z_β_)^2^ *2*σ^2^ / d^2^) was employed to calculate the sample size, and the assumption that the mean difference of D-dimer between the omega-3 treated and untreated patients 1 μg/ml compared to untreated. The population variance was assumed to equal to 2 μg/ml. To detect the assumed mean difference with 95% power at a 5% significance level, minimum of 43 patients is required in each arm.

### Blood sample collection and processing

Blood was drawn under sterile conditions into EDTA coated (2.5 ml) and tri-sodium citrate (2.5 ml) containing vacutainer tubes. The EDTA blood was used for hematological tests. The citrate blood was immediately fractionated into plasma and red blood cells by cold centrifugation at 3000 rpm for 15 min. The resulting top plasma layer was carefully siphoned off, without contaminating with buffy coat, and transferred into another tube and stored at–80 °C. The stored plasma was subsequently used for the determination of coagulation profile and anticoagulation proteins.

### Hematological parameters

Haemoglobin concentration (Hb), haematocrit (Ht), mean corpuscular volume (MCV), mean corpuscular haemoglobin (MCH), mean corpuscular haemoglobin concentration (MCHC), total white blood cell count (TWBC), platelet count (PLTs) and total red blood cell count were measured with the use of Sysmex KX-21 N Automated Hematology Analyzer (Sysmex Corporation, Kobe, Japan).

### Coagulation profile

Prothrombin time (PT) and activated partial thromboplastin time (aPTT) were determined by an automated micro-computer controlled coagulometer, DiaMed-CD2 (DIaMed, GmbH, Cressier, Switzerland). International normalized ratio (INR) was also assessed.

### D-dimer

The concentration of D-dimer was measured by enzyme-linked immune-sorbent assay using i-Chroma hsCRP test kits and i-CHROMA Reader (BodiTech Med Inc., Gang-won do, Korea).

### Anticoagulation proteins C and S

Proteins C and S concentrations were determined by enzyme linked immune-sorbent assay kit **(**Asys Hitech GmbH, Austria).

### Data analysis

According to the parametric or non-parametric distribution, the data were expressed as mean ± SD or median and interquartile range (IQR) as pertinent. The groups were compared for haematological profile, coagulation profile, anticoagulant proteins and D-dimer levels by using one-way an ANOVA on ranks (Kruskal-Wallis H test). When statistical differences were indicated, Dunn’s non-parametric comparison for post-hoc tests were obtained. The statistical significance was assumed at a “p” value of less than 0.05. The data were analysed with SPSS for Windows, Version 19 (SPSS Ltd., Surrey, UK).

## Results

### Demographic and haematological characteristics

The baseline demographic and haematological values of the omega-3 fatty acid treated and untreated patients and healthy controls were described in Table 1. The mean age of the patients on HU was significantly higher than that of the other three groups (*p* < 0.05).

**Table 1:** Baseline demographic and hematological characteristics of the patients and healthy controls

		HbSS Omega −3 treated	HbSS Omega −3 untreated	HbSS HU- treated	HbAA Healthy controls
Number of patients (*n*)		44	52	8	52
	Male	22	28	6	28
	Female	22	24	2	24
Age (years, mean ± SD)		9.8 ± 2.9	10.8 ± 4.0	17.4 ± 0.9*	11.3 ± 4.0

*P < 0.05 hydroxyurea group compared with groups

### Haematological parameters

The hematological values of the groups studied were shown in Table 2. Apart from PLT count, neither treatment with omega-3 nor HU resulted in significant change in haematological profile in comparison to HbSS untreated patients. The haematological parameters MCV, MCH and MCHC values of the patients and healthy controls were not different (*p* > 0.05).

**Table 2:** Mean (±sd) hematological parameter values of omega-3 fatty acid treated, hydroxyurea treated and untreated patients (HbSS) and healthy controls

	Omega-3 FA Treated Patients	Untreated patients	HU Treated	Healthy Controls
Hb (g/l)	75.6 ± 16.2***	75.9 ± 10.3^+++^	79.5 ± 10.3	126.0 ± 10.9
HCt	22.9 ± 4.7***	22.9 ± 3.0^+++^	23.8 ± 2.8	38.0 ± 3.5
RBC	2.8 ± 0.7***	2.7 ± 0.5^+++^	2.8 ± 0.6	4.6 ± 0.5
MCV (fl)	84.3 ± 8.6	86.5 ± 7.1	87.8 ± 11.6	82.3 ± 4.7
MCH (pg)	27.9 ± 3.4	28.6 ± 3.0	29.5 ± 4.4	27.6 ± 1.9
MCHC	33.3 ± 2.0	33.1 ± 1.4	33.4 ± 0.9	33.5 ± 1.5
PLT	489.5 ± 121.1***	533.6 ± 98.7^+++^	414.5 ± 109.9^×^	330.3 ± 72.5
WBC	12.4 ± 4.3***	13.3 ± 3.4^+++^	13.6 ± 3.7	6.3 ± 1.3

***p < 0.001 Omega 3 treated versus healthy controls

++*p* < 0.01, +++*p* < 0.001 Untreated patients versus healthy controls

^x^ *p* < 0.05, HU treated versus untreated patients

### Coagulation profile (PT, aPTT and INR)

In comparison with the healthy controls, the omega-3 fatty acid treated, HU-treated and untreated patients had increased PT, aPTT and INR (*p* < 0.01, Table 3). The omega-3 treated and, HU-treated patients had significantly lower coagulation parameters when compared with Untreated patients (*p* < 0.05, Table 3).

**Table 3:** Mean (±sd) coagulation profile parameter values of omega-3 fatty acid and hydroxyurea treated and untreated sickle cell patients (HbSS) and healthy contols

	Omga-3 treated	untreated	HU treated	Healthy controls
PT (sec)	17.2 ± 1.8^†††^	31.3 ± 11.1^+++^	18.2 ± 1.8^xxx^	14.5 ± 0.7
aPTT (sec)	38.3 ± 4.2^†††^	57.0 ± 11.3^+++^	42.8 ± 2.7^xxx^	37.0 ± 3.8
INR	1.2 ± 0.14^†††^	2.3 ± 0.90^+++^	1.3 ± 0.12^xxx^	1.01 ± 0.05

†††*p* < 0.001 omega-3 treated versus untreated patients

^+++^ *p* < 0.001 untreated patients versus healthy controls

^xxx^ *p* < 0.001 HU treated versus untreated patients

### Plasma D-dimer levels

**Fig. 1:**
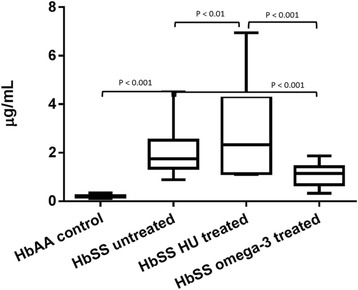
Plasma D-dimer levels of omega-3 fatty acid treated, hydroxyurea treated and untreated patients with homozygous sickle cell disease (HbSS)

The healthy control subjects had a lower level of plasma D-dimer concentration than the omega-3 fatty acid treated, HU-treated and un-treated patients (*p* < 0.001). The omega-3 fatty acid treated group compared with HU-treated (Median = 1.14 (IQR = 0.74) μg/ml vs Median = 2.33.0 (IQR = 3.17) μg/ml, (*p* < 0.001)) and untreated (Median = 1.4 (IQR = 0.74) μg/L vs Median = 1.75 (IQR = 1.16) μg/ml, (*p* < 0.001) patients had a lower plasma D-dimer level. Patients treated with HU had a higher levels of plasma D-dimer compared to HbSS untreated (*p* < 0.01, Fig. 1).

### Plasma proteins C and S levels

**Fig. 2:**
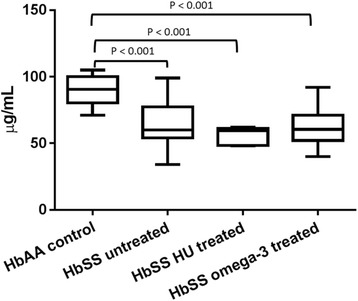
Plasma Protein C levels of omega-3 fatty acid treated, hydroxyurea treated and untreated patients with homozygous sickle cell disease (HbSS) .*There were no significant differences (*p* > 0.05) between the HbSS untreated, HbSS HU treated and HbSS omega-3 treated

The untreated patients had reduced level of plasma protein C compared with the healthy controls (Median = 90.5 (IQR = 20.0) μg/mL, (*p* < 0.001). The omega-3 fatty acid (Median = 60.5 (IQR = 19) μg/mL) and HU (Median = 59.5 (IQR = 13) μg/mL), (*p* > 0.05) treated and the untreated (Median = 60.0 (IQR = 23.0) μg/mL) patients had comparable concentration of protein C (*p* > 0.05, Fig. 2).

**Fig. 3:**
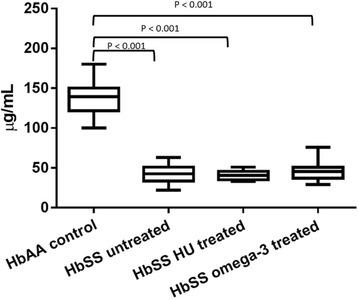
Plasma Protein S levels of omega-3 fatty acid treated, hydroxyurea treated and untreated patients with homozygous sickle cell disease (HbSS). *There were no significant differences (*p* > 0.05) between the HbSS untreated, HbSS HU treated and HbSS omega-3 treated

Plasma protein S concentration was lower in the treated (omega-3 and HU) and untreated patients than in the healthy controls **(**Median = 139.5 (IQR = 28.0) μg/mL, (*p* < 0.001)). There was no difference in protein S level between the unntreated (Median = 42.5 (IQR = 18.0) μg/mL), omega-3 treated (Median = 45.5 (IQR = 14.0) μg/mL) and HU-treated (Median = 40.5 (IQR = 11) μg/mL) patients (*p* > 0.05, Fig. 3).

## Discussion

Hydroxyurea, which is a cytotoxic, antimetabolic and antineoplastic agent, is the only disease-modifying therapy approved for sickle cell disease [14]. Hydroxyurea has been shown to be partially effective in reducing the frequency of vaso-occlusive events; but, there is no evidence that it prevents organ damage [15]. One of the factors which restricts HU usage is that it undergoes renal clearance, and hence there is a need for careful dose adjustment and close monitoring of myelotoxicity in individuals with renal impairment [16]. This vital requirement is hardly possible to undertake in most developing countries where SCD is highly prevalent because of lack of functional facilities and expertise. Therefore, there is a need for safe, effective and easily manageable treatment(s) for children and adult patients with sickle cell disease. Clinical trials have provided evidence that omega-3 fatty acids are effective in reducing frequency and severity of vaso-occlusive episodes, severe anemia, blood transfusion rate, markers of inflammation and oxidative stress [17–21]. In the current study, patients treated with omega-3 showed a significant reduction in D-dimer. The findings of this study indicate that treatment with omega-3 may partially ameliorate SCD-associated coagulopathy.

The SCD patients and healthy controls who participated in this study were homogenous with respect to ethnicity and socio-economic background and the patients received similar quality of care under regular management protocols. The samples were collected in one clinic and at the same time of the year. Therefore, the observed findings are likely to be due to the effect of intervention with omega-3 fatty acids or hydroxyurea treatment rather than of extraneous confounding factors.

Consistent with previous studies, both the omega-3 fatty acid treated and untreated patients had elevated steady state white blood cell and platelet counts confirming that sickle cell disease is a chronic inflammatory disorder [22]. Similarly, Tomer et al. [18] have reported that treatment with omega-3 fatty acids do not effect significantly the blood cell count, MCV or MCHC.

There is evidence which indicates that HU mediates its beneficial effects in SCD, partially, by lowering leukocyte, reticulocyte and platelet counts [23]. In the current study, HU treatment reduced platelet count significantly but had no noticeable effect on WBC. This unexpected effect of HU on WBC among Sudanese children with SCD might be a reflection of the fact that HU is generally being administered mostly to the severely ill children with SCD [24], or a response peculiar to Sudanese SCD patients that warrant further research.

The PT, aPTT and INR levels of the untreated patients were significantly higher than the n-3 fatty acid and hydroxyurea treated groups. Similar findings have been observed on Americans [25] and Nigerians children [26] and adult Jamaicans [6] with sickle cell disease. The prolongation of PT in the adult patients was not as remarkable as in the children. In contrast, another study investigated 17 subjects did not find a difference in mean PT between SCD and healthy children [27]. These controversial findings might be a reflection of the study small sample size.

The mechanism behind the prolongation of PT in children with SCD is not fully understood. It is suggested that impaired liver function [25] and depletion of coagulation factors [28] play a role in the prolongation process. However, it is worth pointing out that a relationship between an abnormal liver function and coagulation prolongation is yet to be established.

In contrast to the findings of the current study, high omega-3 fatty acid intake did not have a significant effect on PT and aPTT in adult patients with sickle cell disease [18], and in adult carcinoma patients undergoing elective surgery [29]. The subjects in the latter two studies, [18, 29], were adults from different ethnic background clinical complications and normal coagulation parameter values at baseline. In addition, the n-3 fatty acids composition of the oil/supplement used in these two studies were different from the high DHA capsules given to the children in the current study. The observed reduction of coagulation parameters in treated SCD patients in this study could be the result of increased availability of the coagulation factors and the possible concomitant reduction in coagulation activation that could surpass its potential detrimental hypo-coagulant effect [30, 31]. HU treatment had a similar effect as n-3 fatty acids on PT and aPTT suggesting that children with abnormal coagulation profile are responsive to either therapy.

The low level steady-state proteins C and S in the Sudanese children with SCD, which agrees with previous findings [6, 32], is in line with the hypothesis that the chronic activation of both the inflammatory and coagulant pathways in SCD are partially due to the disease associated down regulation of anti-coagulant pathway [33]. Protein C, activated by thrombin in the presence of protein S, inhibits the clotting ability of factor V and VIII [34]. The underlying cause of the natural anti-coagulant deficiency is yet to be elucidated. However, it has been generally attributed to the known SCD-associated hemostatic abnormalities and hepatic dysfunction [27]. An earlier study [35] reported a decrease in proteins C in children with sickle cell disease treated with hydroxyurea. That study included 11 children, 5 of them were homozygous SCD patients. The discrepancy of HU effect on proteins C and S between this study and the previous study could be due to the fact that Koc et al. study [35] was relatively underpowered to detect the true HU effect on natural anti-coagulant system.

Despite the observed improvement in coagulation parameters and hypercoagulable state, treatment with n-3 fatty acids did not result in a significant change on the level of the natural anti-coagulant proteins C and S. These findings may indicate that the liver role on low natural anticoagulant in SCD might outweigh the effect of over-consumption due to SCD-associated hypercoagulable state.

The high levels of D-dimer in patients in the current study confirm that hypercoagulability state is one of the major elements of pathophysiology of the disease [33, 36]. Previous studies have reported that omega-3 fatty acid intake is inversely associated with the level of fibrinogen, factor VIII and von Willebrand factor (VWF) [37] and D-dimer [18]. Consistent with the latter study, the current investigation demonstrates high DHA omega-3 fatty acid, but not HU, treatment reduces plasma D-dimer concentration in patients with SCD. The decrease of D-dimer by omega-3 fatty acids has implications for clinical management of patients because plasma D-dimer level is associated with a history of stroke in SCD [36].

This study did not attempt to elucidate the mechanism through which omega-3 fatty acids, particularly DHA and EPA, mediate their anti-coagulant effect. Nevertheless, it is well established that some of the metabolites of these fatty acid are antithrombotic, antiaggregatory, antiinfalammrory and vasodilatory. EPA by competing with arachidonic acid (AA) for cyclooxygenase and lipooxygenase enzymes [38] inhibits the synthesis of the prothrombotic proaggregatory, pro-inflammatory and vasoconstrictor metabolites of AA. Recent animal and human studies suggest that DHA is more potent anti-aggregatory agent than EPA at high doses [39–41]. Interestingly, studies in SCD have demonstrated that endothelial tissue factor expression is specifically dependent upon the nuclear factor-kappa B (NFκB) component of blood mononuclear cells [42]. Our group and others have shown that treatment with high DHA omega-3 fatty acid was associated with down regulation of NFκB gene expression in mononuclear cell and amelioration of SCD-associated chronic inflammatory state [20, 43]. Hence, it is justifiable to attribute the observed improvements in the patient’s hypercoagulable state after high DHA intervention to its suppressive effect on NFκB gene expression and partial resolution of the chronic inflammatory state [44].

Besides the limitations of the observational studies, the current study did not assess liver function in order to have a better understanding of the observed abnormalities of coagulation system and the responses to HU and omega-3 treatments. In addition, the effect of omega-3 fatty acids and HU treatments on markers of thrombin generation such as prothrombin fragment F1, 2 and thrombin-anti-thrombin complexes, was not investigated. Due to the fact that only patients above 10 years of age are treated with HU and generally small fraction of patients were treated with HU in Sudan, we did not manage to recruit enough number of patients on HU matched by age and gender.

## Conclusion

In conclusion, this study provides an evidence that Sudanese children with sickle cell disease have an abnormal coagulating profile which characterized by prolonged PT and aPTT compared to heathy controls. These two parameters were significantly reduced by treatment with either HU or omega-3 fatty acids. With the exception of the D-dimer, treatment with neither omega-3 fatty acids nor HU resulted in a significant change in markers of coagulation compared to the untreated group. To elucidate the putative effects of omega-3 fatty acids on SCD-associated coagulopathy, a well-designed, prospective, randomized trial with an extended panel of hemostaticis is warranted.

## Abbreviations

AA: Arachidonic acid
aPTT: Activated thromboplastin time
DHA: Doxosahexaenoic acid
EPA: Eicosapentaenoic acid
Hb: Hemoglobin
HbAA: Hemoglobin AA
HbSS: Hemoglobin SS
Ht: Haematocrit
HU: Hydroxyurea
MCH: Mean cporpuscular haemoglobin
MCHC: Mean corpuscular haemoglobin concentration
MCV: Mean corpuscular volume
PLt: Platelet count
PT: Prothrombin time
SCD: Sickle Cell Disease
TWBC: Total white blood cell count
